# Design and fabrication of six-volt vertically-stacked GaAs photovoltaic power converter

**DOI:** 10.1038/srep38044

**Published:** 2016-11-30

**Authors:** Yongming Zhao, Yurun Sun, Yang He, Shuzhen Yu, Jianrong Dong

**Affiliations:** 1 Key Lab of Nanodevices and Applications, Suzhou Institute of Nano-Tech and Nano-Bionics, Chinese Academy of Sciences (CAS), Suzhou 215123, P. R. China; 2 University of Chinese Academy of Sciences, Beijing 100049, People’s Republic of China

## Abstract

A six-volt vertically-stacked, high current GaAs photovoltaic power converter (PPC) has been designed and fabricated to produce output power over 1 W under monochromatic illumination. An N^++^-GaAs/P^++^-AlGaAs tunnel junctions (TJs) structure has been used for connecting each sub-cell in this vertically-stacked PPC device. The thickness of the each GaAs sub-cell has been derived based on the calculation of absorption depth of photons with a wavelength of 808 nm using absorption coefficient obtained from ellipsometry measurements. The devices were characterized under non-uniform CW laser illumination at 808 nm with incident power up to 4.1 W. A maximum conversion efficiency of 50.2% was achieved at 0.3 W under non-uniform (coupled in optical fiber) monochromatic illumination, dropping to 42.5% at 4.1 W. The operating voltage at the maximum power point is 5.5–6.0 V, depending on the incident laser power, and an output electrical power output of 1.3 W can be extracted at a laser power of 2.9 W and the maximum electrical power output amounts to 1.72 W. The external quantum efficiency (EQE) measurement indicates that the performance of PPC can be further improved by refining the design of the thickness of sub-cells and improving TJs.

The conversion of monochromatic light into electrical power by photovoltaic power converters (PPCs) has attracted increasing attention^1^,^2^,^3^,^4^,^5^,^6^,^7^,^8^,^9^,^10^,^11^,^12^. This light energy conversion system contain a light source (generally, a laser is employed), a transmission medium (in most cases, an optical fiber) and a PPC^13^,^14^,^15^,^16^,^17^,^18^,^19^. Typical physical process of this technology includes following steps. First, the light energy is transmitted via transmission medium; then, the PPC receives light energy from the transmission medium and convert the optical energy into electrical energy. This energy conversion system can be applied to the place where the electrical energy transmission is inconvenient or is not recommendable. It can take full advantage of not only the properties of fiber (such as electrical insulation and light weight), but also immunity from electro-magnetic, radio frequency (RF), and lightning interferences^20^,^21^,^22^,^23^,^24^,^25^. Therefore, it provides an alternative method and also improves the safety of electricity energy transmission. This system can be applied to many fields, such as sensor, aerospace, explosions, high-voltage power lines, nuclear plants, measurements and medical diagnostic, and so on. It is well known that the transmission loss of near-infrared light (700–870 nm) in fiber is extremely low and high-power near-infrared laser source is commercially. As a results, typically it uses near-infrared light as light sources^1^,^5^,^11^,^14^,^15^,^24^. The bandgap energy of PPCs should be only slightly less than the photon energy of incident light. Therefore, for near-infrared light conversion, GaAs p-n junction material is an excellent choice^3^,^11^,^14^,^26^. The operating voltage of the PPCs is mainly determined by the bandgap of active layer of PPCs and the incident laser power. For a single junction GaAs based PPC, the output voltage is ~1.0 V. However, the most of practical applications require a higher output voltage (typically 5–12 V) to directly drive the connected electronic devices. There are several ways of achieving the required output voltage. The first method is a single junction PPC plus DC-DC converter. However, the DC-DC converter typical only has an efficiency of 80%, and it also has some other limitations in the practical application. The second way is the serially connection of several single junction PPCs (such as pie-shaped PPCs)^14^,^26^. However, the manufacture of serially connected PPCs is relatively complex, and the sub-PPCs should be illuminated with an equal number of photon sources in order to achieve current-match for each sub-PPC. Therefore, the alignment requirement of the incident light for a serially connected PPC system is extremely high. Another feasible approach is the monolithic, multiple junction (MJ) structure^3^,^14^. The vertically-stacked PPC devices can avoid the optical loss and defects caused by etching the isolation trench. This can increase the short-circuit current (J_sc_) and fill factor (FF) of the PPC devices. In addition, the vertically-stacked PPC devices have a lower series resistance than the pie-shaped PPC devices, which improves the FF and open circuit voltage (V_oc_) of the PPC devices. Therefore, the MJ PPC structure has more advantages than the pie-shaped (series connected single junction) PPCs, such as being able to achieve higher conversion efficiency, relaxing the optical alignment requirement of incident light, improving the reliability of device and simplifying the fabrication process^3^,^14^. However, in order to obtain a higher output voltage, more single junction sub-cells are required in the PPC. The conversion efficiency of PPCs with different device structures is shown in Table 1. The maximum conversion efficiency of PPC using single-junction GaAs solar cell is ~60%. Currently, for 4, 5 and 6-V PPC, most of the report adopted pie-shaped structure. The conversion efficiency of this pie-shaped PPC is between 43.5% and 55.1%. The maximum conversion efficiency of 2-junction and 5-junctiom vertically-stacked PPC is 41% and 60%, respectively.

The purpose of this work is to develop a monolithic vertically-stacked multiple junction GaAs laser power converter, with an output voltage over 6 V. Both of theoretically predicted performance of GaAs PPC and experimental results under monochromatic illumination (800–850 nm lasers) are presented. Our results demonstrate the feasibility of a six-volt GaAs PPC with operating voltage ~6 V and output power over 1 W.

## Methods

**Figure 1:**
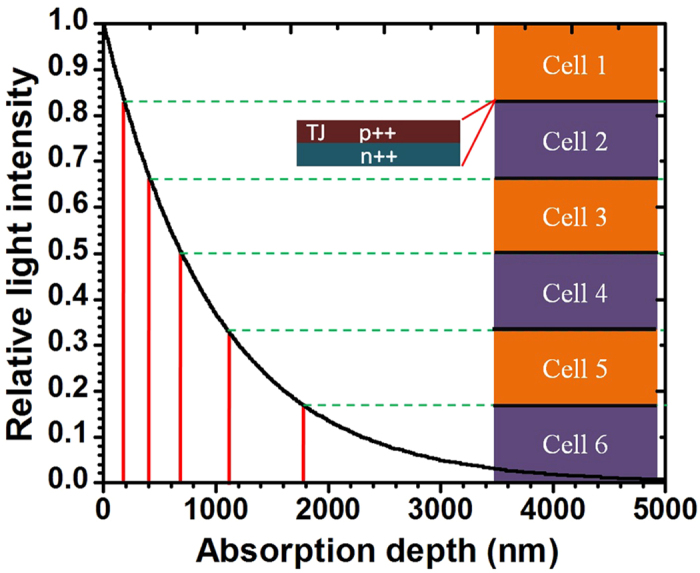
Absorption depth of 808 nm photons in GaAs and schematic of a six-junction GaAs photovoltaic power converter. Where TJ is inserted between every two neighboring sub-cells. The sub-cells are numbered from top to bottom of the device as cell 1 to cell 6.

The simulation and optimization of PPC was conducted by employing the AFORS-Het software. AFORS-HET is an open-source software, which allows to model homo- and hetero- junction optoelectronic devices. In the simulation of PPC devices, the loss mechanisms, such as non-radiative, Shockley-Read-Hall and Auger recombinations were taken into consideration. The optical constants of GaAs and Al_x_Ga_1−x_As (back surface field layer and window layer) material were extracted from the ellipsometry measurements. For each single junction sub-cell, the same ohmic boundary conditions are applied to the top and bottom of them, allowing the calculation of I-V of each sub-cell. An 808 nm light source with full-width at half maximum (FWHM) of 5 nm illuminates the PPC device. In order to avoid current mismatch losses of MJPPCs, the photocurrent must be equally generated in each sub-cell^3^,^14^. The current matching can be realized by adjusting the thickness of each sub-cell. The thickness of the sub-cells have been derived based on the calculation of absorption depth of photons with a wavelength of 808 nm using absorption coefficient obtained from ellipsometry measurements. The absorption loss at the tunnel junctions (TJs) have also been considered. Each sub-cell can be viewed as an individual PPC, absorbing a portion of the input light which passes through the sub-cells above it. The total output voltage is the summation of the output voltage of sub-cells, the number of sub-cells can be chosen to match the voltage required for the application. In this paper, we will focus on the demonstration of vertically-stacked GaAs MJ PPC with a total number of sub-cells of 6. The GaAs sub-cells are interconnected in series via TJs (see Fig. 1). The purpose of the TJ interconnect between the GaAs sub-cells is to provide a low-resistance connection between the P-type back surface field (BSF) layer and the N-type window layer of the adjacent sub-cells.

The PPC samples under investigation were grown on Si-doped (100) GaAs substrates using an AIXTRON 200/4 metal-organic chemical vapor deposition (MOCVD) system with TMAl, TMGa, AsH_3_ as precursors for the growth of the GaAs and AlGaAs epilayers whilst DEZn/CBr_4_ and SiH_4_ were employed as the P and N type dopants, respectively. Prior to the growth of the PPC structure, the GaAs substrates were deoxidized at 700 °C for 5–10 min. The growth temperature was ranged between 600–700 °C and the reactor pressure was 100 mbar. The PPC wafers were processed with conventional photolithography, ohmic contact, etching and isolation. A two-layer antireflection coating (ARC, 90 nm-TiO_2_/60 nm-SiO_2_) was employed which was optimized for the 800–850 nm wavelength range. The P-I-V measurements were performed using two current-carrying probes and a voltage-sense probe on the top contact while the PPC was placed on a temperature controlled test plate. All measurements were conducted at a device temperature of 25 °C.

## Results

**Figure 2:**
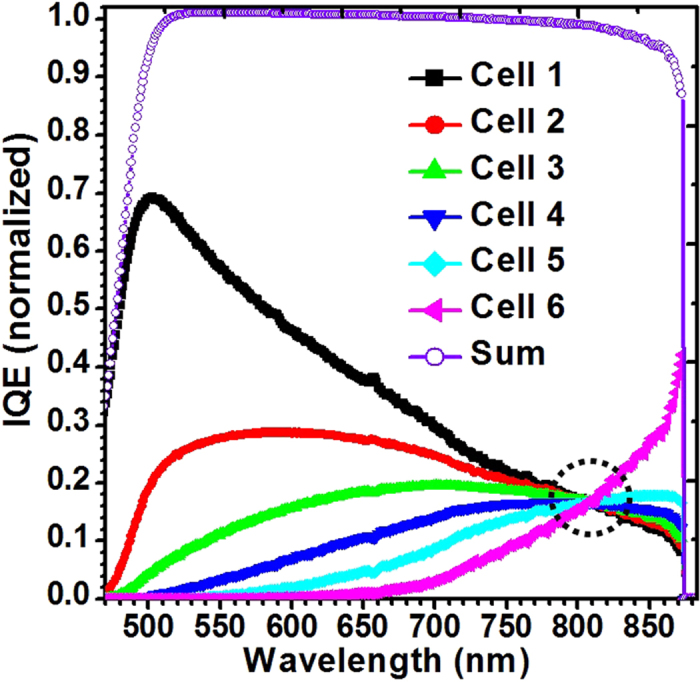
Simulated IQE for sub-cells and the sum of the IQE of all six sub-cells.

The simulated internal quantum efficiency (IQE) curves as a function of wavelength for each of sub-cell and the sum of the IQE of the six sub-cells were shown in Fig. 2. As can be seen, the cell 1 has its maximum IQE at a wavelength of 500 nm. The IQE decreased rapidly for wavelengths below 500 nm is due to the absorption in AlGaAs windows layers. The wavelength-dependent absorption coefficient of photons decreases as wavelength increases. It means that a greater portion of the incident light is transmitted to the bottom cell (i.e. cell 6) at longer wavelength, resulting in the IQE of the top cell (i.e. cell 1) decreases with increasing wavelength. Conversely, the IQE of the cell 6 increases from low values at shorter wavelengths up to a wavelength corresponding to the bandgap energy of GaAs near λ = 870 nm. For the vertically-stacked MJ PPC, the IQE curves of the sub-cells should cross at the designed wavelength (808 nm) by a proper design of sub-cell thickness. In this case, the sum of the IQE of all six sub-cells is roughly a constant at ~98% over the wavelength range of 500–850 nm, which is a similar characteristics to the typical thick GaAs single solar cell.

**Figure 3:**
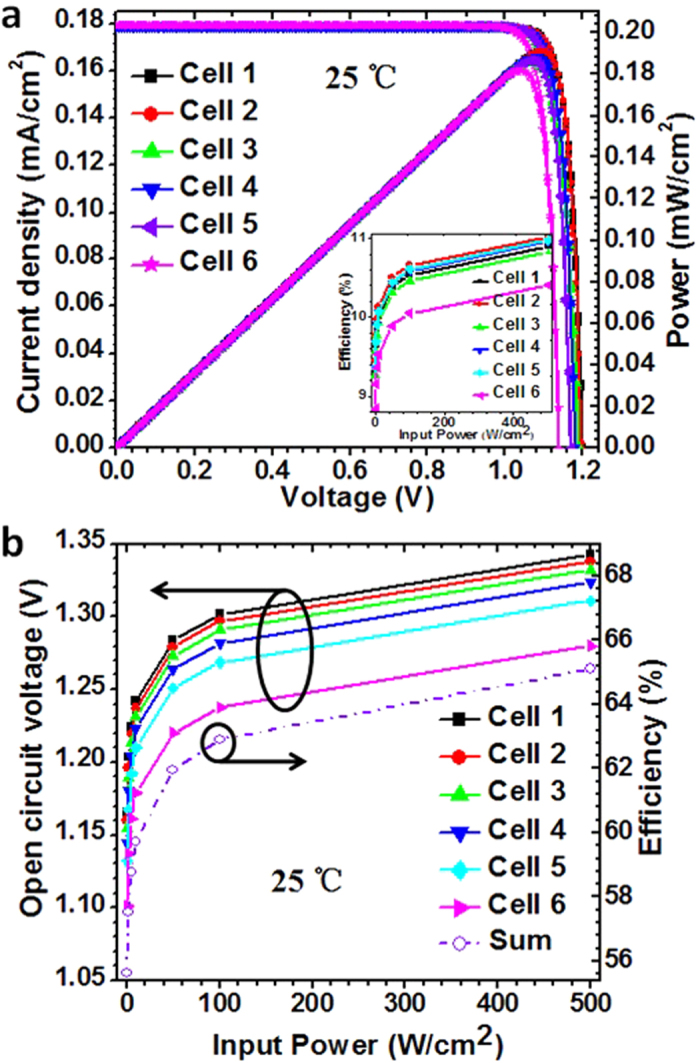
(**a**) Simulated J-V and P-V curves of sub-cells with an incident 808 nm laser power density of 2 W/cm^2^, the inset shows the conversion efficiency of sub-cells under different input laser power densities; (**b**) the open circuit voltage of sub-cells and the total of conversion efficiency of the six sub-cells under different input laser power densities.

The simulated current density-voltage (J-V) and power-voltage (P-V) curves of the sub-cells under monochromatic illumination (808 nm) with an intensity of 2 W/cm^2^ is shown in Fig. 3(a). It can be seen that the current density is nearly matched at the operating point, with a discrepancy less than ±0.2% in J_sc_ between the six sub-cells. These simulation results also demonstrate the diminishing in open circuit voltage (V_oc_) for sub-cells lower in the stack. At an input power of 2 W/cm^2^, the V_oc_ decreases from 1.20 V to 1.14 V from cell 1 to cell 6. The V_oc_ of cell 6 ~5% lower than that of cell 1 is caused by the larger base thickness, leading to the output power of cell 6 nearly 5.2% lower than that of cell 1. The inset shows simulated the conversion efficiency of the sub-cells under different input laser power densities, and the conversion efficiency of the sub-cells firstly increases rapidly and then seems saturated with the increase of input laser power density. The temperature of each sub-cell was set to 25 °C during the simulation. Figure 3(b) shows the V_oc_ of the sub-cells and sum of conversion efficiency of the six sub-cells at different input laser power densities, indicating that the behavior of the V_oc_ and sum efficiency has a similar trend. The optimal conversion efficiency of six-junction PPCs and average V_oc_ of sub-cells increases from 55.6% to 65.1% and 1.15 V to 1.32 V, respectively, as input laser power density increasing from 0.5 W/cm^2^ to 500 W/cm^2^.

**Figure 4:**
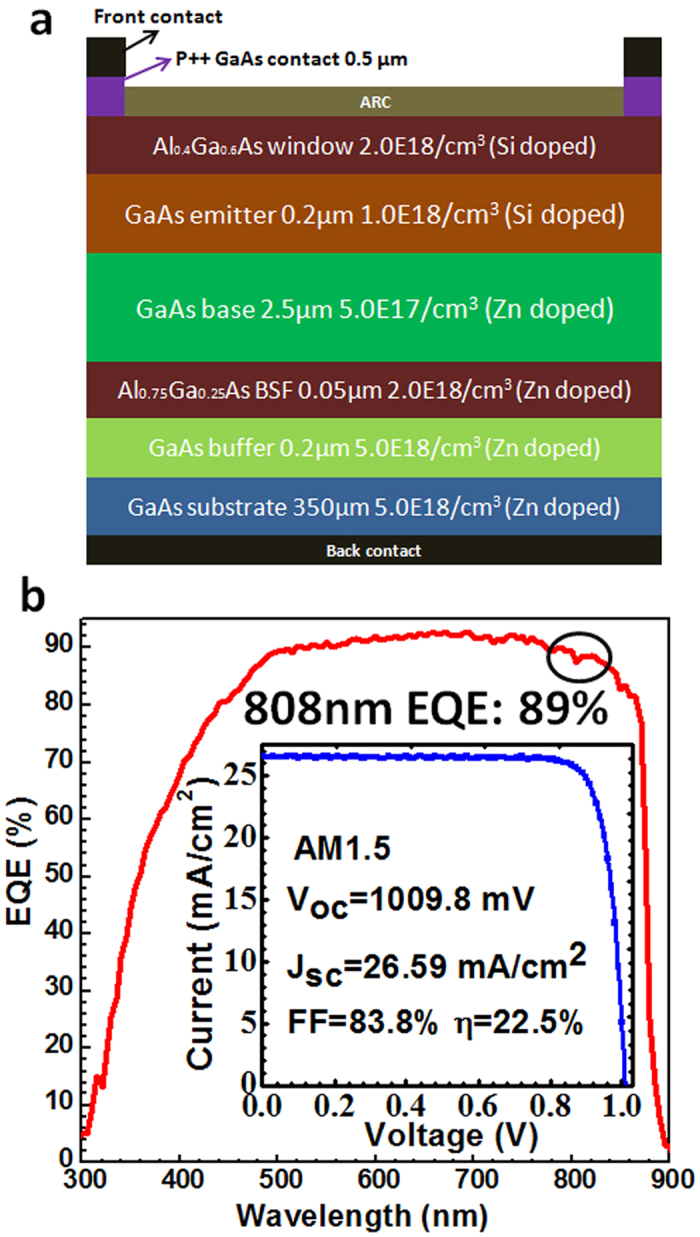
(**a**) Cross-section of the GaAs single junction solar cell, the inset shows the picture of this solar cell; (**b**) EQE of this GaAs solar cell, the inset shows its J-V characteristics.

Prior to the growth of six-junction PPCs, a GaAs single junction solar cell structure was firstly designed and grown as a reference. The schematic cross section of this GaAs single junction solar cell is shown in Fig. 4(a). The active region of the device consists of a GaAs P-N junction (base/emitter), covered by the top window layer (Al_0.4_Ga_0.6_As) and the bottom BSF layer (Al_0.75_Ga_0.25_As). The P-type doping density of the base layer is 5.0 E17/cm^3^ while the N-type doping density of the emitter layer is 1.0 E18/cm^3^. The current density-voltage (J-V) characteristics of the solar cell were measured under the AM 1.5D spectrum using an ABET sun 2000 solar simulator. The measured result is depicted in Fig. 4(b). The calibration at one-sun showed that the GaAs single junction solar cell had a photovoltaic conversion efficiency of 22.5% with an V_oc_ of 1.009 V, a short circuit current density (J_sc_) of 26.59 mA/cm^2^, and a FF of 83.8%. The lower J_sc_ of solar cell is due to the fact that the ARC and the top electrode has not been optimized. The J_sc_ could be increased by optimizing the ARC and the top electrode.

External quantum efficiency (EQE) measurements were carried out to give the wavelength-dependent photovoltaic response. The EQE of the GaAs single solar cell is about 89% at the wavelength of 808 nm. The reflectance of this GaAs solar cell is about 5%. Therefore, the GaAs single junction solar cells are able to achieve IQE in excess of 90%, attesting to the proper quality of the material and device structure. Due to the division of the available photons among the six sub-cells, and therefore an IQE peak large than 15% should be expected for this vertically-stacked PPC device structure.

There are several reasons for output power loss for this vertically-stacked device structure, and the most important one is the failure of optically transparent connection between the sub-cells called as “TJs”. Ideally, the TJs should be made of material with a bandgap equal to or larger than that of the sub-cells to avoid absorption within the TJs. In addition, for a TJ the tunneling current and voltage drop are also important parameters. It is necessary for both of N-type and P-type semiconductor to be doped in excess of 1.0 E19/cm^3^ to acquire high tunneling current. However, high-bandgap materials are often have difficulties in N-type doping to these levels, leading to a relatively low peak tunneling current (J_peak_) and limiting the output power of the PPCs.

**Figure 5:**
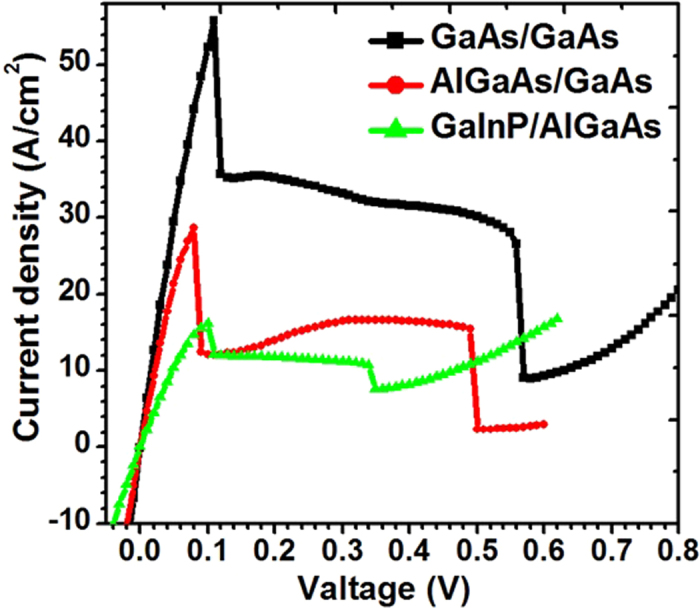
J-V characteristics of N++-GaAs/P++-GaAs, N++-GaAs/P++-AlGaAs and N++- GaInP/P++-AlGaAs TJs.

Therefore, three kinds of test TJs (N++-GaAs/P++-GaAs, N++-GaAs/P++-AlGaAs and N++- GaInP/P++-AlGaAs) were designed and manufactured for PPC devices on n-type GaAs substrates, and their J-V characteristics were shown in Fig. 5. The thickness of each heavily-doped (N^++^ or P^++^) layer is 25 nm. The doping levels in the tunnel junction themselves are estimated to be 1.0 E20/cm^3^ and 1.0 E19/cm^3^, for the p and n-type layers, respectively. The TJs were capped with a 200 nm of GaAs C-doped to 1.0 E20/cm^3^ to achieve ohmic contacts with a low resistance. For the growth of TJs, the CBr_4_ and SiH_4_ were employed as the P and N type dopants, respectively. The growth temperature of Si-doped layer (such as n++GaAs and n++InGaP) is maintained at 700 °C. However, in order to obtain a higher doping level, the growth temperature of C-doped layer (such as p++GaAs and p++AlGaAs) is decreased to 600 °C. It should be noted here that in order to acquire a higher doping level, a δ-doping method is employed for Si-doped layer. The J_peak_ of N++-GaAs/P++-GaAs, N++-GaAs/P++-AlGaAs and N++-GaInP/P++-AlGaAs TJ is 55.8, 28.6 and 16.1 A/cm^2^, respectively. While the voltage drop of the TJ at peak current density is 0.11 V, 0.08 V and 0.10 V, respectively. Unfortunately, the N++-GaAs/P++-GaAs TJ has a strong optical absorption at 808 nm. Considering the above-mentioned parameters and the practical problem in the material growth of TJs, an N++-GaAs (20 nm)/P++-AlGaAs (25 nm) TJ structure has been used in the vertically-stacked PPC devices, and this choice ensure not only a high peak tunneling current density, but also a less loss due to absorption.

A six-junction PPC structure was grown based on the simulated and experimental results. The growth conditions of the sub-cells are similar to the single junction GaAs solar cell. Each GaAs sub-cell consists of 20 nm P-Al_0.75_Ga_0.25_As back surface field (BSF) (doping 2.5 E18/cm^3^), P-GaAs base (doping 5.0 E17/cm^3^), N-GaAs emitter (doping 1.0 E18/cm^3^) and 45 nm N-Al_0.4_Ga_0.6_As window (doping 6.0 E18/cm^3^, except the sub-cell 1). The thickness (base+emitter) of cell 1, 2, 3, 4, 5 and 6 is 120 nm, 140 nm, 175 nm, 270 nm, 430 nm and 1900 nm, respectively. The N++-GaAs (20 nm)/P++-AlGaAs (25 nm) TJ structure was used to connect sub-cells. On the top of cell 1, a 1 μm-Al_0.4_Ga_0.6_As window layer was grown as the current spreading layer. A 75 nm-N^++^ GaAs cap-layer enables excellent ohmic contact with the top electrode grid, and the GaAs cap-layer is selectively etched away between the grid lines prior to the fabrication of the ARC to eliminate optical absorption. The reflectivity of PPCs at wavelength of 808 nm decreases from 20% to 0.2%.

**Figure 6:**
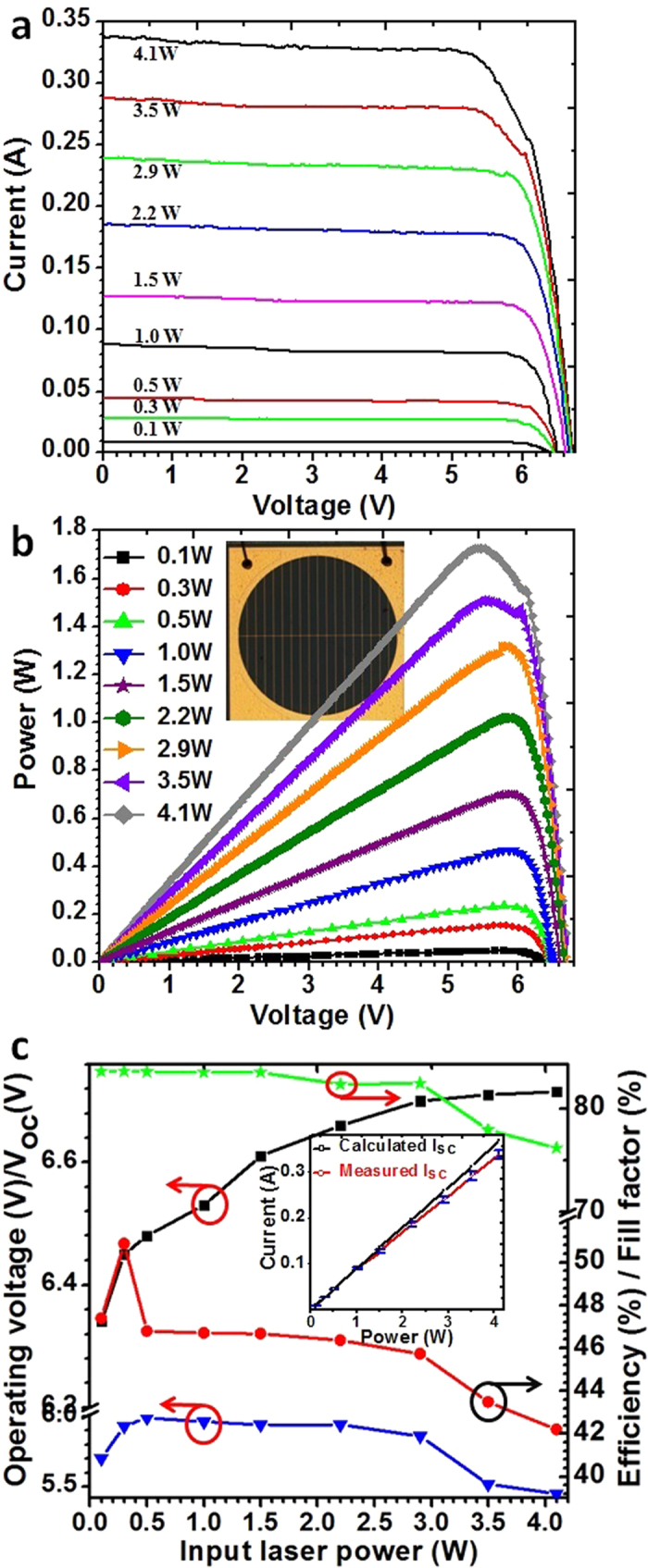
(**a**) I-V characteristics of the PPC device (the device with designated illumination area of 11.34 mm^2^) under different incident laser powers, (**b**) output power of the PPC device versus incident laser power (808 nm fiber-coupled laser source), the inset shows a picture of PPC. (**c**) open circuit voltage(square), operating voltage (triangle), conversion efficiency (circle) and fill factor (star)of PPC device as a function of the incident laser power; The inset shows the summary of calculated and measured I_sc_.

The I-V characteristics and output power of the PPC device under different incident laser powers are shown in Fig. 6(a,b). The measurements were performed under fiber-coupled 808 nm laser illumination. As can be seen, the PPC device exhibits a linear current response to input laser power. A slight shunt-like characteristics visible in these curves is attributed to a slight current mismatch among sub-cells, and this is confirmed by EQE measurement. A current mismatch increases the FF, however, the resulted current losses reduce the conversion efficiency^27^. The operating voltage at the maximum power point is 5.5–6.01 V, depending on the incident laser power (see in Fig. 6(c)), and an electrical output power of 1.3 W can be extracted at an input laser power of 2.9 W. The maximum electrical power output amounts to 1.72 W.

The V_oc_, FF, and the conversion efficiency of PPC device as a function of the incident laser power are shown in Fig. 6(c). It can be seen that the V_oc_ increases almost logarithmically with the incident laser power up to 3.5 W. The V_oc_ increasing from 6.28 V to 6.70 V as incident laser power increases from 0.05 W to 2.9 W. At higher incident laser power, the V_oc_ does not increase further as expected. One reason is the PPC devices heats up during the I-V measurement which causes the band gap shrinkage and therefore the V_oc_ drop^14^. In fact, the accurate measurement of the V_oc_ under continuous and higher-intensity laser light illumination is difficult due to gradual heating of the PPC devices.

The high FF is due to the current mismatch^27^ and almost keep constant as the incident laser power increasing from 0.05 W to 1.5 W. As incident laser power increases further, the FF reduces drastically, and this can be explained by the increase of series resistance loss at higher current generated at higher illumination intensity^3^ and the current limited by the TJs at higher incident laser power (when incident laser power is at/above 3.5 W). The annealing of the TJs during the growth of PPC structure was found to produce a significant decrease in the peak current density of TJs^28^,^29^,^30^,^31^. This figure also shows the conversion efficiency vs incident laser power, the PPC device shows the conversion efficiency of 50.2% at 0.3 W, dropping to 42.5% at 4.1 W. Several factors cause the flattening of the conversion efficiency curve, However, the most important two are the spectral effect and thermal effect^3^,^4^. A higher laser power increases the cavity temperature of laser source, resulting in the laser spectral beam of multi-peaked, multi-modal and a non-Gaussian profile^3^. In addition, at higher illumination intensities, the PPC devices also have the problem of heat dissipation. These two factors led to a lower conversion efficiency at a higher incident laser power.

We calculated the I_sc_ (short circuit current) should be generated in theoretically based on the measured EQE. The inset shows the summary of calculated and measured I_sc_. At lower incident optical power (<1.0 W), the theoretical and experimental results consistent with each other. However, at higher incident optical power (>1.0 W), the measured I_sc_ gradually lower than theoretical predicted. This is more in line with the actual situation. At higher incident laser power, the PPC devices would have a higher junction temperature, resulting in the EQE of PPC devices decreasing. The power stability of the laser source is ±3%. Thus, the I_sc_ also has an error of ±3%.

**Figure 7:**
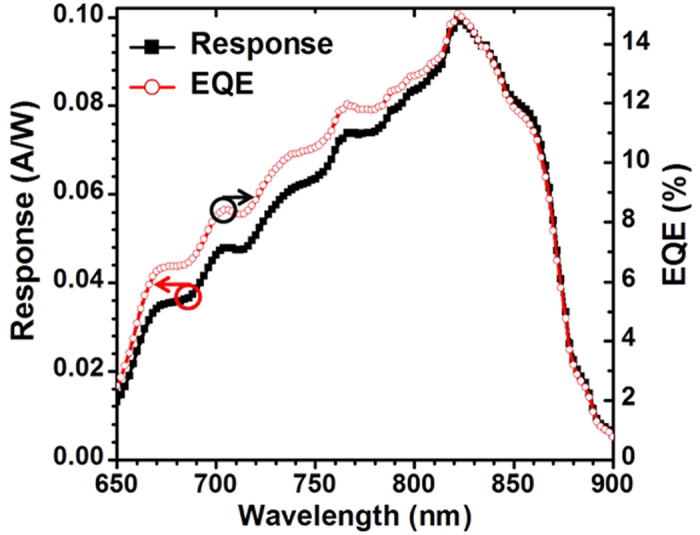
The measured EQE and spectral response of the six-junction PPC device.

Figure 7 shows the measured EQE/spectral response (SR) of a typical six-junction PPC device. The maximum EQE of the designed PPC device occurs at a wavelength of 808 nm. However, the fabricated PPC device reaches the maximum EQE of ~14.9% at a wavelength of 822 nm. There is a current mismatch at the wavelength 808 nm. The EQE is ~13.3% and ~14.9%, respectively, at wavelength of 808 nm and 822 nm. The SR is ~0.087 A/W and ~0.099 A/W, respectively, at wavelength of 808 nm and 822 nm. It is expected that a change of the 808 nm laser to 822 nm could lead to a 12.8% gain in current generation and therefore, an increase in conversion efficiency 1.1 times in comparison with 808 nm-monochromatic illumination. It can be expected that an EQE of above 15% nm can be achieved at wavelength of 808 nm when optimizing the thickness of each sub cell and using GaInP/AlGaAs TJs instead of GaAs/AlGaAs ones. In addition, the performance of TJs can be greatly improved by inserting a thin layer of GaAs (3 nm)^32^.

## Conclusion

The simulation and experimental results of six-volt vertically-stacked, GaAs MJ PPCs were presented. The simulation was used to understand the effects of PPC structural parameters on the performance of the PPC. The devices were characterized under CW laser illumination at 808 nm with incident power densities up to 4.1 W. A very high current density has been produced with over 1 W of output power under monochromatic illumination. A conversion efficiency of 50.2% was achieved at 0.3 W under 808 nm laser illumination, dropping to 42.5% at 4.1 W. The operating voltage at the maximum power point is 5.5–6.0 V, depending on the incident laser power, and an output electrical power of 1.3 W was obtained at a laser power of 2.9 W. The maximum electrical power output amounts to 1.72 W. We expect the conversion efficiency to be further improved by refining the design of the thickness of sub-cells and improving TJs.

## Additional Information

**How to cite this article**: Zhao, Y. *et al*. Design and fabrication of six-volt vertically-stacked GaAs photovoltaic power converter. *Sci. Rep.* **6**, 38044; doi: 10.1038/srep38044 (2016).

**Publisher's note:** Springer Nature remains neutral with regard to jurisdictional claims in published maps and institutional affiliations.

## Figures and Tables

**Table 1:** The conversion efficiency of PPCs with different device structures.

Type	Voltage (V)	Incident energy	Illumination wavelength (nm)	Efficiency(%)
GaAs	1	5 W/cm^2^	808	52.2^1^
GaAs	1	36.5 W/cm^2^	810	54.9^15^
GaAs	1		809	~60^33^
GaAs	1	43 W/cm^2^	810	53.4^34^
GaAs	1	0.522 W	835	56^17^
Vertically-stacked	2	100 W/cm^2^	810	41^34^
Pie-shaped	2	15 W/cm^2^	810	~47.5^14^
Pie-shaped	4	0.8 W	830	~47^35^
Pie-shaped	4	8.5 W/cm^2^	810	55.1^14^
Pie-shaped	4	0.17 W	808	45.4^36^
Pie-shaped	5	2 W/cm^2^	793	50.4^5^
Vertically-stacked	5	11 W/cm^2^	835	60^3^
Pie-shaped	6	17 W/cm^2^	810	43^34^
Pie-shaped	6	22 W/cm^2^	810	42.7^14^
Pie-shaped	6	~0.25 W	808	~43.5^36^
Vertically-stacked	6	2.6 W/cm^2^	808	50.2*

^*^Means our result.
